# The effect of emotion recognition and mindfulness on depression symptoms: A case-control study

**DOI:** 10.1192/j.eurpsy.2022.259

**Published:** 2022-09-01

**Authors:** O. Aydın, S. Tvrtkovic, E. Çakıroğlu, P. Ünal-Aydın, A. Esen-Danacı

**Affiliations:** 1 International University of Sarajevo, Psychology, Ilidza, Bosnia and Herzegovina; 2 Celal Bayar University, Psychiatry, Manisa, Turkey

**Keywords:** Depression, symptom severity, Emotion recognition, Mindfulness

## Abstract

**Introduction:**

Abnormalities in emotion recognition (ER) are frequently reported in depression, with lowered recognition accuracy in patients with major depressive disorder (MDD) when compared to healthy individuals. Mindfulness was found to directly impact the severity of depressive symptoms, by negative cognition and dysfunctional reaction recognition.

**Objectives:**

The aims of this study were to compare ER and mindfulness levels between MDD patients and healthy controls (HC), as well as to examine whether ER and mindfulness are related to symptom severity in MDD patients.

**Methods:**

68 patients with MDD and 93 HC participated in the study. A sociodemographic form, Reading the Mind in the Eyes Test (RMET), Five Facet Mindfulness Questionnaire-Short Form (FFMQ-S) and the Montgomery Asperg Depression Scale (MADRS) were administered. Group comparison in ER and mindfulness was assessed using the Multivariate analysis of covariance (MANCOVA). Bivariate correlations and multiple linear regression analyses were performed to assess the associations between depression severity, ER and mindfulness in the patient group.

**Results:**

Better ER and higher levels of mindfulness were found in HCs relative to the MDD group. A positive association between depression severity and the non-reactivity facet of mindfulness was found, indicating that in the MDD group non-reactivity was a significant predictor for depression severity. On the other hand, ER was not significant in predicting symptom severity.

**Conclusions:**

Non-reactivity, unlike other dimensions of mindfulness, seems to increase with the severity of depressive symptoms among MDD patients. To particularly focus on this subdimension in mindfulness techniques may yield better outcomes in alleviation of depressive symptoms.

**Disclosure:**

No significant relationships.

